# Ultra-coherent nanomechanical resonators based on inverse design

**DOI:** 10.1038/s41467-021-26102-4

**Published:** 2021-10-01

**Authors:** Dennis Høj, Fengwen Wang, Wenjun Gao, Ulrich Busk Hoff, Ole Sigmund, Ulrik Lund Andersen

**Affiliations:** 1 Center for Macroscopic Quantum States (bigQ), Department of Physics, Technical University of Denmark, Fysikvej, 2800 Kgs. Lyngby, Denmark; 2 Department of Mechanical Engineering, Technical University of Denmark, Niels Koppels Allé, 2800 Kongens Lyngby, Denmark; 3 State Key Laboratory of Disaster Reduction in Civil Engineering, Tongji University, Shanghai, 200092 China

**Keywords:** Mechanical properties, Nanoscale devices, Design, synthesis and processing

## Abstract

Engineered micro- and nanomechanical resonators with ultra-low dissipation constitute a promising platform for various quantum technologies and foundational research. Traditionally, the improvement of the resonator’s performance through nanomechanical structural engineering has been driven by human intuition and insight. Such an approach is inefficient and leaves aside a plethora of unexplored mechanical designs that potentially achieve better performance. Here, we use a computer-aided inverse design approach known as topology optimization to structurally design mechanical resonators with optimized performance of the fundamental mechanical mode. Using the outcomes of this approach, we fabricate and characterize ultra-coherent nanomechanical resonators with, to the best of our knowledge, record-high *Q* ⋅ *f* products for their fundamental mode (where *Q* is the quality factor and *f* is the frequency). The proposed approach - which can also be used to improve phononic crystals and coupled-mode resonators - opens up a new paradigm for designing ultra-coherent micro- and nanomechanical resonators, enabling e.g. novel experiments in fundamental physics and extreme sensing.

## Abstract (web summary)

Nanomechanical resonators can be improved through rational design. Using an inverse design approach the authors achieve ultra-coherent resonators and pave the way towards the engineering of high performance micro- and nanomechanical resonators.

## Introduction

Topology optimization is a computational morphogenesis procedure widely applied in engineering to determine the best possible structural design and material distributions within a prescribed design domain by maximizing a set of performance targets^1^. Examples include the maximization of the structural stiffness of an object under certain design and manufacturing constraints to determine the optimal design of a full-scale aeroplane wing^2^ or a girder of a suspension bridge^3^, and the maximization of light concentration to develop the optimal design of nanophotonic resonators^4^.

The basic strategy of topology optimization is to define a design domain in which material can be distributed. Material is being added to or removed from this domain, and founded on a physical model for the system, a gradient-based computational method is used to optimize the figure-of-merit. Through iterations, material is gradually redistributed towards the optimal design for which the figure of merit is either maximized or minimized, depending on the problem to be solved.

We use topology optimization to design a nanomechanical resonator towards maximizing its *Q* ⋅ *f* (*Qf*) product^5–7^. Previously, improving the resonator’s performance has been done through a combination of human intuition and trial-and-error based on experience and approximative analytical expression for the different dissipation mechanisms of the resonator^8–17^. Such an intuition-based approach has recently led to impressive progress in increasing the *Qf* product of mechanical resonators by using a combination of dissipation dilution^11^, soft-clamping^15^, thin-clamping^18^, and strain engineering^16^. Despite these recent successes, the approach inevitably leaves out many, possibly counter-intuitive, designs that might exhibit superior behavior. Topology optimization counteracts this problem as it directly develops the optimized structure under given initial design constraints and loss models with no geometrical pre-assumptions.

## Results

### Model and topology optimization

**Fig. 1:**
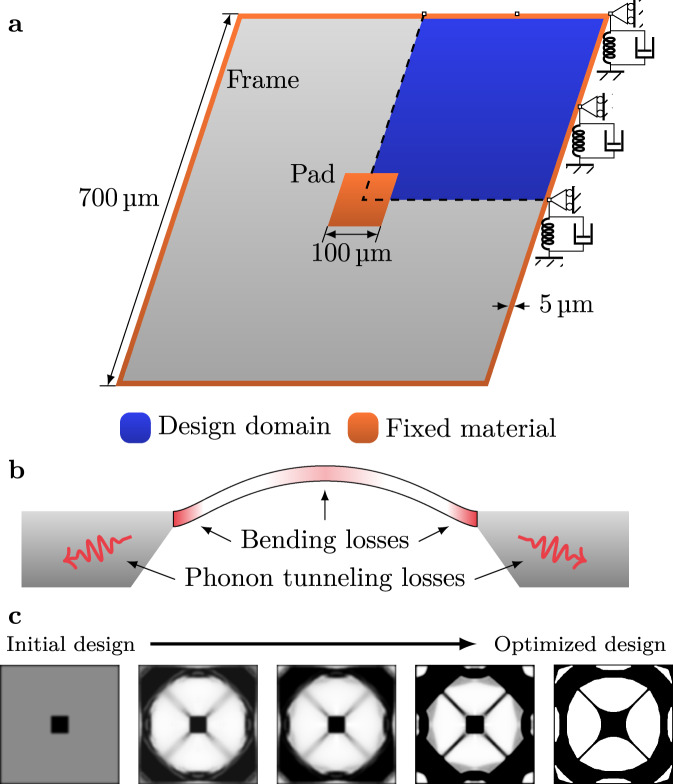
Topology optimization model. **a** Illustration of the model used in topology optimization. Note the springs illustrate a continuous distribution of springs. **b** Illustration of the two damping mechanisms: intrinsic losses in the form of bending and phonon tunneling losses. **c** Illustration of the optimization procedure of resonator D1 with snapshots of the design evolution. The degree of transparency indicates the material density.

Aiming at maximizing the *Qf* product of the fundamental mode of a nanomechanical resonator suitable for opto-mechanical experiments, we consider the initial structure illustrated in Fig. 1a. It comprises an area of 700 × 700 μm^2^ and a thickness of 50 nm with a single central pad of size 100 × 100 μm^2^ made of pre-stressed silicon nitride (that allows for the interaction with light via radiation pressure force^19,20^) and a narrow frame of 5 μm to ease fabrication. The remaining space is free to evolve through topology optimization. The pre-stressed resonator is numerically discretized with 200 × 200 quadrilateral shell elements using the finite element method. In the topology optimization procedure, a design variable is assigned to each element to control the material occupation in the element which is to be iteratively updated. Design robustness with respect to manufacturing errors is enforced by simultaneously considering three design realizations (eroded, normal, and dilated, corresponding to over-etched, nominal, and under-etched realizations of the design) which are maximized for their minimum *Qf* product. Meanwhile, the fundamental frequency is constrained to be equal or above a prescribed value to ensure structural connectivity, and a volume fraction is used to constrain the resonator weight and regularize the optimized resonator shape. The deterministic, adjoint-based optimizations are initiated by a uniform design variable distribution, satisfying the volume constraint and typically require a total of 2000 finite element evaluations to converge. Detailed descriptions of the topology optimization procedure are included in the Methods section.

**Fig. 2:**
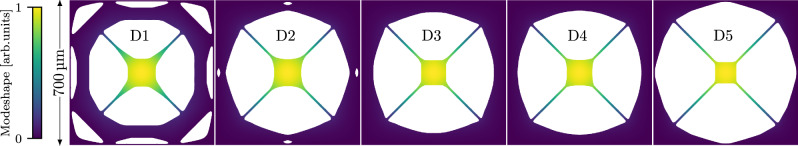
Designs overview. Overview of topology optimized structures and the mode shape of their respective fundamental mode. Design D1 and D5 are optimized assuming only bending losses or phonon tunneling losses, respectively. Design D2–D4 assume different weighted combinations of the two damping mechanisms.

Two damping mechanisms associated with intrinsic losses and phonon tunneling losses have been included in the model (Fig. 1b). The intrinsic losses are modeled as bending losses in the form of hysteretic damping, i.e. using a lossy Young’s modulus. The phonon tunneling loss (PTL) associated with radiation of the phonons into the substrate was modeled by coupling the boundary out-of-plane displacements to continuously distributed lossy springs (illustrated in Fig. 1a) while keeping all the other degrees of freedom fixed. Since the loss mechanisms are not yet completely understood, we perform optimizations for five different ratios of intrinsic and PTL losses, with loss values estimated from the physically realized reference design [8] such that the reference resonator under the five loss models exhibits a similar *Q*-value as the experimental result. The optimized designs based on the five different loss models are denoted as D1–D5. For D1 and D5 the system is purely limited by intrinsic loss and by phonon tunneling loss, respectively, while for D2–D4 the ratio is gradually changed. The exact ratios can be found in the Methods section. To illustrate the iterative procedure of the topology optimization, in Fig. 1c we show the evolution of the design of resonator D1. The final topology optimized designs for all five cases are illustrated in Fig. 2. The images have been slightly filtered in post-processing with the aim of removing buckling-prone features and smoothing sharp features to prevent high tensile stresses at the boundaries (see Methods section).

Interestingly, the optimized design of D1 is similar to the membrane design suggested and experimentally tested in Beccari et al.^21^ but using a completely different approach. They arrive at this geometry based on considerations on soft-clamping using a hierarchical design concept^17^.

### Fabrication and characterization

**Fig. 3:**
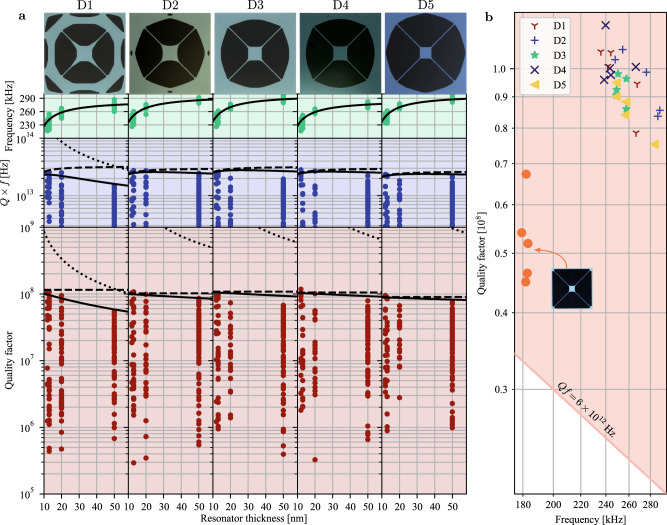
Results overview. **a** Overview of the measured frequencies and quality factors across all designs and thicknesses together with selected microscope images. Solid lines correspond to theory fitted to the measured frequencies and best attained quality factors. Dotted and dashed lines are associated with the phonon tunneling and intrinsic loss contributions, respectively. For some designs, the theory curves for the phonon tunneling loss is not visible on the plots. **b** Quality factor plotted against frequency for the five best samples for each design. The shaded area marks the parameter regime in which the resonator may undergo quantum coherent oscillations at room temperature. The inset is the nominal trampoline design fabricated in this work and used as a reference^13^. The large spread in the Q factors is due to the uncontrollable coupling of the fundamental mode to the substrate mode. In the *Qf* plot, we only include measurements of membranes for which the *Q* factor was above 10^7^ but all measurement data are included in the plots for the frequency and *Q* factor.

**Fig. 4:**
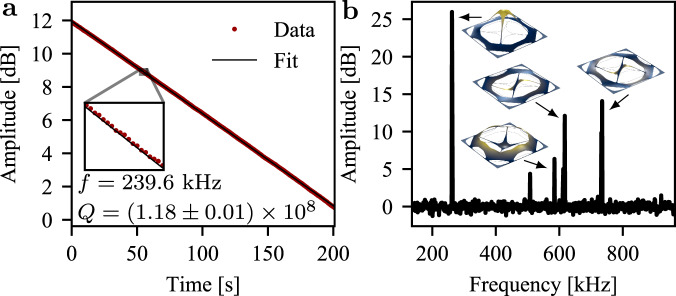
Resonator result. **a** Mechanical ring-down measurement of the best measured sample corresponding to design D4. **b** Spectrum of a D1 sample.

The post-processed designs were patterned on high-stress (*σ*_0_ ≤ 1.2 GPa) silicon nitride with a thickness of 12–50 nm grown by low-pressure chemical vapor deposition on a silicon wafer. We release the resonators by back-etching the silicon substrate in a window of 1.4 × 1.4 mm^2^. The fabricated structures are shown in Fig. 3a. To measure the mechanical frequency and quality of the fundamental mode, ring-down measurements were carried out in vacuum (pressure <10^−7^ mbar) at room temperature using high-sensitivity fiber-based homodyne detection (see Methods section). An example of a ring-down measurement of a fundamental mode of frequency 240 kHz exhibiting an amplitude ring-down time of ~160 s is illustrated in Fig. 4a. This corresponds to a *Q* factor of 1.18 ± 0.01 × 10^8^ and a *Qf* product of 2.83 × 10^13^ Hz. We also present an example of a thermal noise spectrum including some higher-order modes in Fig. 4b.

We performed ring-down measurements of the fundamental mode of 967 devices that include all the topologically optimized resonators, D1–D5, as well as the conventional non-optimized trampoline resonator^13,14^ which is used as reference structure. A collection of our measurements on frequency, quality factor, and *Qf* product is presented in Fig. 3. It is clear from these measurements that the topologically optimized resonators are superior to the reference trampolines and that they are all deeply into the regime where the resonator is able to undergo coherent oscillations (corresponding to *Qf* > 6 × 10^12^ Hz) as required for quantum coherent experiments^19^.

**Fig. 5:**
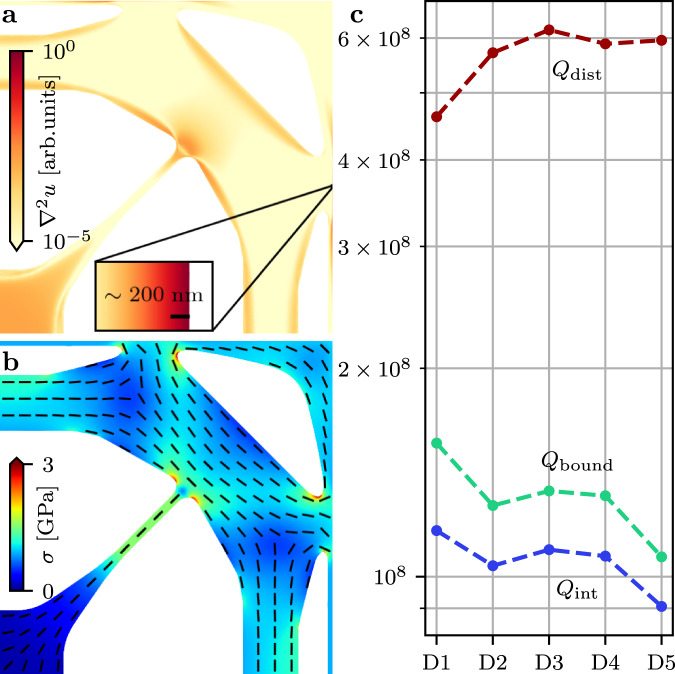
Design analysis. **a** Bending loss distribution of D1 on a logarithmic scale. The inset highlights the high bending losses at the boundary. **b** Static von Mises distribution. The bars indicate the direction of the first principal stress component. **c** Numerically predicted intrinsic quality factor *Q*_int_ partitioned into boundary (*Q*_bound_) and distributed (*Q*_dist_) bending losses with ${Q}_{{{{{{{{\rm{int}}}}}}}}}^{-1}={Q}_{{{{{{{{\rm{bound}}}}}}}}}^{-1}+{Q}_{{{{{{{{\rm{dist}}}}}}}}}^{-1}$.

To understand the limitations of their performance, we fit our best attained results to a theory for the intrinsic and phonon tunneling losses. As the intrinsic loss Δ*W* is mainly dominated by the clamping losses near the boundaries, we directly estimate these losses from the expression

1 $${{\Delta }}W=\int \frac{\pi \phi }{12}\frac{E{h}^{3}}{1-{\nu }^{2}}{\left(\frac{{\partial }^{2}u}{\partial {x}^{2}}+\frac{{\partial }^{2}u}{\partial {y}^{2}}\right)}^{2}\ {{{{{{{\rm{d}}}}}}}}x{{{{{{{\rm{d}}}}}}}}y$$

where *h*, *E*, *ν* are the thickness, Young’s modulus and Poisson’s ratio of the resonator material^15^. *u*(*x*, *y*) is the mode shape and the loss angle is modeled as *ϕ* = 1/(*hβ*) where *β* is related to the intrinsic damping at the surface. The mode profiles of all designs are simulated using the COMSOL Multiphysics package with the results shown in Fig. 5.

Phonon tunneling losses are simulated by placing a distribution of independent lossy springs along the outer boundary with only one degree of freedom in the out-of-plane direction defined by a lossy imaginary stiffness per unit length $k^{\prime}$. These two loss contributions (intrinisic and phonon tunneling losses) are then adjusted to match the best experimental data using the loss-dependent factors *β* and $k^{\prime}$ as fitting parameters. We find *β* = (2.93 ± 0.19) × 10^11^ m^−1^ and $k^{\prime} =(4.09\pm 1.36)\times 1{0}^{12}\ {{{{{{{\rm{N}}}}}}}}/{{{{{{{{\rm{m}}}}}}}}}^{2}$, and the resulting theory curves for all designs are shown in Fig. 3a where dotted (dashed) lines correspond to phonon tunneling (intrinsic) losses while the total contribution is represented by solid lines. It is clear that the best performing resonators of all five designs are mainly dominated by intrinsic losses. However, for some resonators we observe markedly lower performance (with *Q* factors below 10^7^) which we attribute to a near-resonant coupling to the substrate modes, consequently leading to significantly higher phonon tunneling losses which eventually become the dominating loss factor. This randomized coupling to the substrate modes can be circumvented by inserting a damping shield encapsulating the resonator^22^.

The fitting of the loss models to the results of the best performing membranes yields a guidance to the appropriateness of the calibration parameter used to develop the five designs. For example, design D5 was developed under the assumption that phonon tunneling loss dominates which contradicts the result of the fitting procedure as it concludes that intrinsic losses are dominating. It is thus more likely that the calibration parameters used for designs D1–D2 are more appropriately describing the physical system as the assumed loss ratios for these designs are qualitatively reminiscent of those attained via the fitting procedure.

We highlight the source of intrinsic losses by plotting the bending loss distribution of design D1 in Fig. 5a. First we note that there is a significant amount of bending loss near the boundaries (as highlighted by the inset) and near the intersection between the circular frame and the tethers. The latter dilutes the bending loss of the former (resulting from the strong mode confinement) and is ﻿likely the origin of the quality enhancement . It is similar to the effect observed in resonators based on fractal structures^21^. The observed bending loss at the central pad is due to its low stress leading to a locally reduced stiffness and consequently, sharper bending. In Fig. 5b we illustrate the stress distribution from which we observe a large stress component on the circular frame perpendicular to the tether. The wavelength predicted by the stress and frequency is ~2 mm which is larger than the dimensions of the resonator. Therefore, it cannot exist on the circular frame resulting in mode confinement and dilution of losses. Finally, we compared the amount of boundary bending losses (localized along the outer boundary) to the estimated amount of distributed bending losses (far away from the boundary) as shown in Fig. 5c. It is clear that the resonator is limited by the boundary losses.

## Discussion

Micromechanical oscillators with a *Qf* product of >10^13^ Hz for the fundamental mode will have a number of intriguing applications in quantum optomechanics and precision sensing. One of the main requirements in quantum optomechanics, e.g. for cooling the oscillator to the quantum ground state, interrogating macroscopic quantum superpositions, and entangling different systems, is that the decoherence time exceeds the mechanical oscillation period. This translates into the requirement that *Qf* > *k*_B_*T*/*ℏ* = 6 × 10^12^ Hz at room temperature (where *k*_B_ is Boltzman’s constant, *ℏ* is the reduced Planck’s constant, and the temperature is *T* = 300 K)^19,20^. While most of the resonators fulfill this requirement, our best performing device yields around 4 coherent oscillations which is the largest number ever reported for the fundamental mode of a membrane at room temperature. Our devices will also exhibit exceptional performance in force sensing measurement as for example used in magnetic resonance force microscopy of electron and nuclear spins^23^. In such measurements the sensitivity is limited by the thermal noise ($\sqrt{4m{k}_{{{{{{{{\rm{B}}}}}}}}}T\frac{2\pi f}{Q}}$ where *m* is the mass) which we find to be at 10 ${{{{{{{\rm{aN}}}}}}}}/\sqrt{{{{{{{{\rm{Hz}}}}}}}}}$ for the best devices which is significantly beyond what is attainable with currently available room temperature force microscopes.

The topology optimization method, that we have here employed to maximize the *Qf* product of the fundamental mode of a membrane, is applicable to many other similar morphogenesis problems in engineering of high-performance micro- and nanomechanical resonators. It can for example be applied to finding the optimal structure for maximizing the dissipation dilution effect - and thus the *Qf* product – in phononic crystal resonators where *Q* factors of nearly one billion and *Qf* products of >10^15^ have already been achieved without topology optimization^15,16,24^. This can be achieved by running the optimization algorithm over the higher-order modes (rather than the fundamental modes as done in this work). Another interesting avenue for new studies using our methodology is to optimize other application-specific parameters instead of the *Qf* product. An example is the optimization of the co-operativity parameter associated with the coupling of a specifically functionalized mechanical oscillator to spins^25^, light^26^ or charges^27^ with the aim of significantly enhancing quantum transduction or sensing. Finally, it is also possible to optimize structures with additional constraints, either structural constraints enabling certain applications or parameter constraints, e.g. fixing the mass to a large value with the aim of maximizing the coupling to gravity as required for example for interrogating the quantum nature of gravity^28,29^. Our methodology thus has the potential to revolutionize the way nano- and micro-mechanical systems are being designed enabling radically new applications and fundamental explorations.

## Methods

We employed a density-based topology optimization approach^1^ to design ultrahigh coherent resonators. The basic methodology and the detailed optimization formulation are described in the following.

Pre-stressed membrane resonators are simulated using finite element methods with the 4-node MITC (Mixed Interpolation of Tensorial Components) quadrilateral shell element^30^. The mechanical dynamic problem is solved in two steps: (1) Establish static equilibrium of a pre-stressed membrane resonator under prescribed stress; (2) Identify resonating modes using linear eigenvalue analysis. The FE equations are stated in discrete form as,

2 $${{{{{{{{\bf{K}}}}}}}}}_{0}{{{{{{{{\bf{U}}}}}}}}}_{0}={{{{{{{{\bf{F}}}}}}}}}_{0}$$

3 $$\left({{{{{{{{\bf{K}}}}}}}}}_{0}+{{{{{{{{\bf{K}}}}}}}}}_{\sigma }\left({{{{{{{{\bf{U}}}}}}}}}_{0}\right)+i{{{{{{{\bf{C}}}}}}}}-{\omega }_{j}^{2}{{{{{{{\bf{M}}}}}}}}\right){{{{{{{{\boldsymbol{\phi }}}}}}}}}_{j}={{{{{{{\bf{0}}}}}}}}.$$

Here **F**_0_ is the equivalent force vector resulting from a prestress *σ*_0_, **K**_0_ represents the linear stiffness matrix and ${{{{{{{{\bf{K}}}}}}}}}_{\sigma }\left({{{{{{{{\bf{U}}}}}}}}}_{0}\right)$ represents the initial stress stiffness matrix that depends on the displacement **U**_0_ of the prestress problem in Eq. (2). **C** and **M** denote damping and mass matrices, *ω*_*j*_ and **ϕ**_*j*_ are the angular frequency and modal profile of the *j*-th resonating mode and $i=\sqrt{-1}$ is the imaginary unit.

The damping matrix, **C**, covers intrinsic and phonon tunneling losses. The intrinsic losses are considered via a relaxation mechanism described by a complex-valued Young’s modulus $\tilde{E}=\left(1+i{\eta }_{s}\right)E$. The phonon tunneling losses are modeled using damped springs distributed along the boundary with a total stiffness of ${\bar{k}}_{b}=\left(1+i{\eta }_{b}\right){k}_{b}$ and *k*_*b*_ = 8.315 × 10^7^ kN/m^2^. The detailed calculation formulations of quantities in Eqs. (2) and (3) can be found in Gao et al.^7^. The quality factor and frequency of the *j*-th resonating mode are calculated by

4 $${Q}_{j}=\frac{\Re \left({\omega }_{j}\right)}{2\Im \left({\omega }_{j}\right)},\quad {f}_{j}=\frac{\Re \left({\omega }_{j}\right)}{2\pi }.$$

In the density-based topology optimization approach, an element-wise design variable, ${x}_{e}\in \left[0,\,1\right]$, is introduced to indicate the material occupation in element *e*. To avoid checkerboard pattern and mesh dependence^1^ and enhance design discreteness, the design variables are first filtered using a density filter^31^ and then smoothly projected using a hyperbolic tangent threshold function^32^, given as

5 $${\tilde{x}}_{e}=\frac{\mathop{\sum}\nolimits_{k\in {N}_{e}}{w}_{e}({{{{{{{{\bf{y}}}}}}}}}_{k}){v}_{k}{x}_{k}}{\mathop{\sum}\nolimits_{k\in {N}_{e}}{w}_{e}({{{{{{{{\bf{y}}}}}}}}}_{k}){v}_{k}}$$

6 $${\bar{x}}_{e}=\frac{\tanh \left({\beta }_{1}\eta \right)+\tanh \left({\beta }_{1}\left({\tilde{x}}_{e}-\eta \right)\right)}{\tanh \left({\beta }_{1}\eta \right)+\tanh \left({\beta }_{1}\left(1-\eta \right)\right)}.$$

Here, ${\tilde{x}}_{e}$ is the filtered design variable, **y**_*k*_ are the center coordinates of element *k*. *v*_*k*_ and *x*_*k*_ are the corresponding volume and design variable of element *k*, respectively. *N*_*e*_ is the neighborhood of element *e* within a certain filter radius specified by ${N}_{e}=\left\{k| \ \parallel {{{{{{{{\bf{x}}}}}}}}}_{k}-{{{{{{{{\bf{y}}}}}}}}}_{e}\parallel \le r\right\}$, and *w*_*e*_(**y**_*k*_) = *r* − ∥**y**_*k*_ − **y**_*e*_∥. ${\bar{x}}_{e}$ is the projected design variable of element, *e*. When *β*_1_ is large, ${\bar{x}}_{e}\approx 1$ if ${\tilde{x}}_{e} \, > \, \eta$ representing Si_3_N_4_, and ${\bar{x}}_{e}\approx 0$ if ${\tilde{x}}_{e} \, < \, \eta$ indicating void. The projection suppresses gray element density regions induced by the density filter when *β*_1_ is sufficiently large and ensures black-white designs when the optimization converges. Moreover, it mimics the manufacturing process and the manufacturing errors can be taken into accounts in the optimization by choosing different thresholds, *η*, as discussed later.

The Young’s modulus of element *e* is directly related to the projected design variable using the Rational Approximation of Material Properties (RAMP)^33^ and the mass density is linearly interpolated as

7 $${E}_{e}=\frac{{\bar{x}}_{e}}{1+q\left(1-{\bar{x}}_{e}\right)}(E-{E}_{0})+{E}_{0},\quad q=3$$

8 $${\rho }_{e}={\bar{x}}_{e}\left(\rho -{\rho }_{0}\right)+{\rho }_{0}.$$

Spurious modes caused by inappropriate stiffness-to-mass ratios in low-density regions are suppressed by setting *E*_0_ = 10^−6^*E* and *ρ*_0_ = 10^−7^*ρ* to represent void in this study. Wrinkling-like instabilities in low-density regions are alleviated using a displacement interpolation with detailed formulations presented in Gao et al.^7^.

To enhance the design robustness with respect to manufacturing errors and impose a minimal length scale in the nominal design, a three-case robust formulation is employed^32^. Three design realizations are generated to mimic an eroded, normal, and dilated manufacturing processes. The optimization problem for designing ultrahigh coherent resonators is formulated to maximize the *Qf* product of the fundamental mode for the worst case of the three design realizations, subjected to frequency constraints and a volume fraction constraint, given as

$$\begin{array}{ll}\mathop{\max }\limits_{{{{{{{{\bf{x}}}}}}}}}&\mathop{\min }\limits_{\eta }\qquad {{{{{{\mathrm{ln}}}}}}}\,\left({Q}_{1}\left({{{{{{{\bf{x}}}}}}}},\eta \right){f}_{1}\left({{{{{{{\bf{x}}}}}}}},\eta \right)\right)\\ s.t.&{f}_{1}\left({{{{{{{\bf{x}}}}}}}},\eta \right) \, > \,{f}^{* }\\ &\frac{{{{{{{{{\bf{v}}}}}}}}}^{T}\bar{{{{{{{{\bf{x}}}}}}}}}\left({{{{{{{\bf{x}}}}}}}},{\eta }_{d}\right)}{\mathop{\sum}\limits_{e}{v}_{e}}\le {v}^{* }\\ &{{{{{{{\bf{0}}}}}}}}\le {{{{{{{\bf{x}}}}}}}}\le {{{{{{{\bf{1}}}}}}}}\\ &\eta \in \left\{{\eta }_{e},{\eta }_{i},{\eta }_{d}\right\}\end{array}$$

The three design realizations are generated using $\eta \in \left\{0.55,\,0.5,\,0.45\right\}$ with a filter radius of *r* = 15 μm. This corresponds to a minimal feature size of 6.7 μm in both solid and void regions of the nominal design. The prescribed frequency lower bound and volume fraction upper bound are f^*^ = 240 kHz and v^*^ = 0.5.

Gradients of the objective and constraint functions are calculated using the adjoint sensitivity analysis and the chain rules^7,31,32^. The design variables are iteratively updated using the deterministic mathematical programming approach, Method of Moving Asymptotes (MMA)^34^ based on the gradients of the objective and constraints. *β*_1_ is updated until the convergence criterion is satisfied by ${\beta }_{1}^{(n+1)}=1.1{\beta }_{1}^{(n)}$ reaching a maximum value of 120.

The loss parameters used in the five design cases, (D1, D2, D3, D4, D5), are *η*_*s*_ = (2.500; 1.790; 1.120; 0.515; 0.000) × 10^−4^ and *η*_*b*_ = (0.000; 0.095; 0.190; 0.285; 0.380) calibrated against the reference trampoline design.

**Fig. 6:**
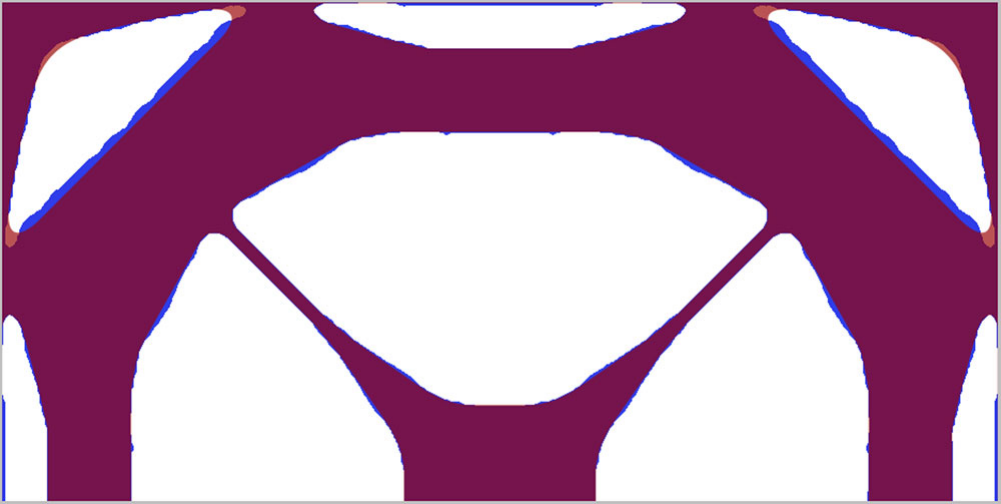
Design post-processing. Comparison of the generated topology optimized design (blue) and the subsequent post-processed design (red) overlaid on top used in experiments for design D1.

The design outputs of the optimization algorithm have some irregular shape features caused by the particular density-based topology optimization approach that we are applying. As mentioned above, in this approach, an elemental density variable (taking values between 0 and 1) indicates the material occupation in the finite elements (0 for void and 1 for silicon nitride) of the design, thereby producing a gray-scaled evolution design that ideally converges towards a white/black design (corresponding to the density variable being 0 or 1). Due to finite element discretization using quadrilateral shell elements, the final designs possess stair-cases and a very small amount of gray values. This may lead to local tensile stress, and would eventually result in breakage during fabrication. To avoid this, we spatially filtered the designs in post-processing by removing convex features followed by low-pass filtering using a rectangular window with a 5 μm width, thereby smoothing the edges as illustrated in Fig. 6.

We deposit stoichiometric silicon nitride onto a 100 mm single-crystal silicon wafer of 500 μm thickness using low-pressure chemical vapor deposition. This is followed by spincoating photoresist onto the wafer and transfer of the different resonator designs using UV-lithography. The photoresist is developed and the silicon nitride is etched in these regions by means of reactive ion etching. Residual photoresist is removed using oxide plasma, and finally, the trampolines are released in potassium hydroxide at 80 °C followed by cleaning in hydrochloric acid and sulfiric acid mixed with ammonium persulfate.

Measurements of the frequency and quality factor of the resonators are performed using optical interferometry driven by a laser with a wavelength of 1550 nm. The laser beam is reflected off the vibrating membrane (located inside a vacuum chamber at low pressure < 10^−7^ mBar), and the resulting phase shift is detected with high-sensitivity using a phase-locked homodyne detector and recorded with a spectrum analyzer. Excitation of the mechanical oscillator is done by modulating the intensity of the laser at the resonance frequency. Once excited, the modulation is switched off and the amplitude decay is subsequently measured. We ruled out the potential effect of photothermal-induced modifications of the *Q* factor by conducting the *Q* value measurements with a variety of different power levels without observing any changes.

**Fig. 7:**
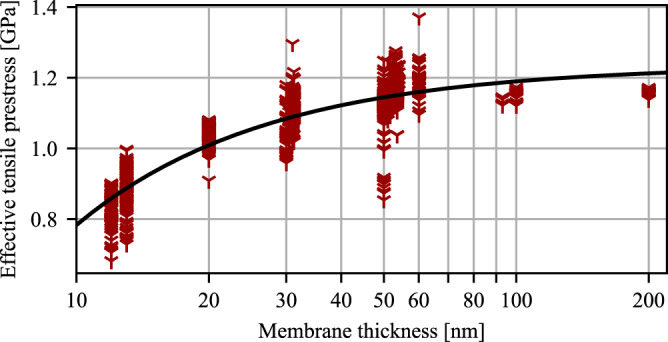
Stress versus thickness. Extraction of the stress-thickness relation of stoichiometric silicon nitride based on frequency measurements of 2573 samples.

To model the frequency dependency of the silicon nitride thickness in Fig. 3a, the tensile prestress dependency of the thickness is needed. The stress-thickness dependency is believed to be caused by the oxidization layer which introduces a compressive stress contribution onto the silicon nitride film dependent on its thickness^35^. Assuming that the oxidized layer is much smaller than the total film thickness, we model the effect by the expression *σ*(*h*) = *σ*_0_ − *β*_*σ*_/*h* where *σ*_0_ is the asymptotic prestress parameter and *β*_*σ*_ is a coefficient that determines how fast the prestress changes with thickness. We fit these two parameters against data attained from the measurement of tensile prestress from 2573 samples of different thicknesses as shown in Fig. 7. The tensile stress was derived by measuring the resonance frequency and comparing to predicted values from finite element simulations noting the $f\propto \sqrt{\sigma }$ dependency. This approach has some inherent uncertainties related to fabrication and the assumptions of the material parameters of silicon nitride. We find *σ*_0_ = 1.235 ± 0.002 GPa and *β*_*σ*_ = 4.52 ± 0.06Pa ⋅ m.

## Supplementary information

Peer Review File

## Data Availability

The data generated in this study have been deposited in the figshare data repository database 10.11583/DTU.14394254.
