# Supplementary material for: Ultra-coherent nanomechanical resonators based on inverse design
Source: Nat Commun. 2021 Oct 1;12:5766. doi: 10.1038/s41467-021-26102-4 (PMC8486777; doi:10.1038/s41467-021-26102-4)
Supplement: Supplementary file 1 — Peer Review File [file 41467_2021_26102_MOESM1_ESM.pdf]

nature portfolio

Peer Review File

Ultra-coherent nanomechanical resonators based on inverse design

Open Access This file is licensed under a Creative Commons Attribution 4.0 International License, which permits use, sharing, adaptation, distribution and reproduction in any medium or format, as long as you give appropriate credit to the original author(s) and the source, provide a link to the Creative Commons license, and indicate if changes were made. In the cases where the authors are anonymous, such as is the case for the reports of anonymous peer reviewers, author attribution should be to 'Anonymous Referee' followed by a clear attribution to the source work. The images or other third party material in this file are included in the article's Creative Commons license, unless indicated otherwise in a credit line to the material. If material is not included in the article's Creative Commons license and your intended use is not permitted by statutory regulation or exceeds the permitted use, you will need to obtain permission directly from the copyright holder. To view a copy of this license, visit http://creativecommons.org/licenses/by/4.0/.

Editorial Note: This manuscript has been previously reviewed at another journal that is not operating a transparent peer review scheme. This document only contains reviewer comments and rebuttal letters for versions considered at *Nature Communications*.

REVIEWERS' COMMENTS

Reviewer #1 (Remarks to the Author):

I would like to thank the authors for the thorough revision of the manuscript.

My only remaining concern is that the the manuscript should explain what the difference is between the three design realisations eroded, normal, and dilated.

Otherwise it can be published in Nature Communications.

REVIEWERS' COMMENTS

Reviewer #1 (Remarks to the Author):

I would like to thank the authors for the thorough revision of the manuscript.
My only remaining concern is that the the manuscript should explain what the difference is between the three design realisations eroded, normal, and dilated.
Otherwise it can be published in Nature Communications.

Response

Thanks for the positive feedback. A clearer explanation of the three designs as mentioned have now been included.
